# Breast Delineation in Full-Field Digital Mammography Using the Segment Anything Model

**DOI:** 10.3390/diagnostics14101015

**Published:** 2024-05-15

**Authors:** Andrés Larroza, Francisco Javier Pérez-Benito, Raquel Tendero, Juan Carlos Perez-Cortes, Marta Román, Rafael Llobet

**Affiliations:** 1 Instituto Tecnológico de la Informática, Universitat Politècnica de València, Camino de Vera s/n, 46022 València, Spain; alarroza@iti.es (A.L.); fjperez@iti.es (F.J.P.-B.); rtendero@iti.es (R.T.); jcperez@iti.es (J.C.P.-C.); 2 Department of Epidemiology and Evaluation, IMIM (Hospital del Mar Research Institute), Passeig Marítim 25-29, 08003 Barcelona, Spain; mroman@parcdesalutmar.cat

**Keywords:** mammography, breast segmentation, segment anything model (SAM)

## Abstract

Breast cancer is a major health concern worldwide. Mammography, a cost-effective and accurate tool, is crucial in combating this issue. However, low contrast, noise, and artifacts can limit the diagnostic capabilities of radiologists. Computer-Aided Diagnosis (CAD) systems have been developed to overcome these challenges, with the accurate outlining of the breast being a critical step for further analysis. This study introduces the SAM-breast model, an adaptation of the Segment Anything Model (SAM) for segmenting the breast region in mammograms. This method enhances the delineation of the breast and the exclusion of the pectoral muscle in both medio lateral-oblique (MLO) and cranio-caudal (CC) views. We trained the models using a large, multi-center proprietary dataset of 2492 mammograms. The proposed SAM-breast model achieved the highest overall Dice Similarity Coefficient (DSC) of 99.22% ± 1.13 and Intersection over Union (IoU) 98.48% ± 2.10 over independent test images from five different datasets (two proprietary and three publicly available). The results are consistent across the different datasets, regardless of the vendor or image resolution. Compared with other baseline and deep learning-based methods, the proposed method exhibits enhanced performance. The SAM-breast model demonstrates the power of the SAM to adapt when it is tailored to specific tasks, in this case, the delineation of the breast in mammograms. Comprehensive evaluations across diverse datasets—both private and public—attest to the method’s robustness, flexibility, and generalization capabilities.

## 1. Introduction

Breast cancer represents a significant global health challenge. Mammography is the primary imaging modality used for breast cancer screening and is associated with a significant decrease in breast cancer mortality. Ongoing technological advancements have yielded improved imaging features, facilitating the early detection of pathological signs associated with the disease [1].

Percent density (PD) refers to the relative amount of fibroglandular tissue within the breast area compared to the overall breast area. Advanced computational methods, including deep learning algorithms, have been employed to improve the accuracy of breast density estimation from mammograms. These methods analyze digital mammograms to classify and segment dense and fatty tissues, providing a quantifiable measure of breast density. This quantification is crucial as breast density is associated with breast cancer risk [2], and its precise assessment can enhance cancer detection and screening effectiveness [3,4].

One fundamental task to estimate the PD is the correct delineation of the breast tissue, excluding other areas that are not of interest that appear in the mammogram, such as background, text, pectoral muscle, and/or abdomen. Once the breast region is isolated, quantitative analysis can be performed to assess various characteristics, such as breast density. Knowledge of the breast contour also allows for further analysis of breast abnormalities such as bilateral asymmetry. Standardizing the breast segmentation process helps ensure consistency and reliability in breast tissue analysis. It is essential in large-scale screening programs where multiple radiologists or automated systems may be involved in interpreting mammograms [5].

Previous breast delineation methods incorporate threshold-based segmentation, morphological manipulation, and a combination of Hough transforms and texture features [6,7]. Sansone et al. [8] analyzed two popular packages that have been proven robust against various situations and were suitable for pectoral muscle removal. Gudhe et al. [3] proposed a new deep learning architecture that automatically estimates the area-based breast percentage density from mammograms using a weight-adaptive multitask learning approach, which improves the baseline traditional methods. The pectoral muscle is mainly seen in medio lateral-oblique (MLO) views. For this reason, most of the available tools are aimed at the pectoral muscle exclusion in MLO views rather than in cranio-caudal (CC) views in which the muscle is sometimes present.

In our previous study [9], we introduced a fully automated framework for dense-tissue segmentation. It included breast detection, pectoral muscle exclusion, and dense-tissue segmentation. The dense-tissue segmentation step was implemented using a Convolutional Neural Network (CNN) architecture named CM-Ynet. However, breast detection and exclusion of the pectoral muscle were carried out using traditional image processing methods. While these methods generally performed well, they encountered challenges in detecting the pectoral muscle in CC images and excluding unwanted regions, such as the abdomen. In response to these challenges, this work introduces a deep learning-based method for breast region segmentation, with a specific focus on enhancing pectoral muscle removal in CC views.

The Segment Anything Model (SAM), released in 2023 by META AI, represents a significant advancement in image segmentation. As the largest model of its kind, the SAM has been trained on billions of labeled images, covering a vast array of object types and scenarios. This extensive training enables the SAM to perform segmentation tasks on nearly any object, regardless of its complexity, without additional specific training. The SAM operates by using prompts or seed points, which can be either positive (to specify areas for segmentation) or negative (to exclude certain areas). Additionally, the SAM can utilize a bounding box approach, segmenting all objects within the specified area [10]. While the SAM is a state-of-the-art research advancement in natural image segmentation, it does not perform satisfactorily when directly applied to medical image segmentation. Recent research suggests that this limitation primarily arises from the significant domain gap between natural images and medical images [11,12].

The main focus of current research is to adapt the SAM to various medical image segmentation tasks. Previous studies have explored different ways to fine-tune the SAM for specific datasets or modalities, such as retinal, abdominal, and cardiac images [13,14,15,16]. Wu et al. [17] proposed the Medical SAM Adapter, which incorporates medical knowledge into the SAM using a simple adapter technique, and tested it on 19 segmentation tasks. More recently, a thorough study on using the SAM for medical 2D images was introduced, distinguished by its extensive data collection, detailed analysis of fine-tuning options, and comprehensive performance evaluation [18]. However, none of these studies addressed the breast segmentation problem.

In this paper, we investigate and evaluate the SAM for breast delineation using a large-scale multi-center dataset that aims to remove all non-breast tissue in MLO and CC views, thereby providing a robust preprocessing method for further mammogram evaluations. We compare our proposed SAM-breast model with traditional and deep learning-based breast segmentation approaches, evaluating their performance on both proprietary and publicly available datasets.

## 2. Materials and Methods

### 2.1. Datasets

We used five datasets for this study, two proprietary and three publicly available.

- GVA (proprietary): A multi-center dataset that covers 11 medical centers of the Generalitat Valenciana (GVA) as part of the Spanish breast cancer screening network. It includes 1785 women with ages from 45 to 70. The CC and MLO views were available for 10 out of 11 of the centers, while one center only collected the CC view. This dataset was used for training, validation, and testing. The dataset was randomly partitioned into 75% (2492 mammograms) for training and validation (10%) and 25% for testing (844 mammograms). All the mammograms are of the type “for presentation”. Further details about this dataset can be found in our previous work [9].
- IMIM (proprietary): A dataset composed of 881 images obtained at the Hospital del Mar Research Institute (IMIM). It was included solely for testing purposes to better evaluate the generalization performance of the models. This dataset consists of images from three different acquisition devices. One of these devices (Hologic Lorad Selenia, Marlborough, MA, USA) is older and was used to obtain images back in 2012. As a result, the image quality is lower, making the segmentation task more challenging. Only CC views were provided for this dataset, and they are also of the type “for presentation”.
- CSAW-S (public): The CSAW-S dataset, released by Matsoukas et al. [19], is a companion subset of CSAW [20]. This subset contains mammograms with expert radiologist labels for cancer and complementary labels of breast anatomy made by non-experts. The anonymized dataset contains mammograms from 150 cases of breast cancer, some of them including both MLO and CC views. We generated the breast masks for our experiments by combining the provided mammary gland and pectoral muscle labels, thus obtaining a total of 270 images with breast mask segmentations.
- InBreast (public): A well-known publicly available dataset [21]. It has ground truth annotations for the pectoral muscle in MLO views. We used these annotations to generate the ground truth breast mask for a total of 200 images.
- Mini-MIAS (public): The Mini-MIAS database [22], created by the Mammographic Image Analysis Society (MIAS), is a resource that has been extensively used in prior research. It contains 322 digitized mammographic films in MLO view. The breast masks that we used for evaluation were obtained from Verboom et al. [23].

**Figure 1:**
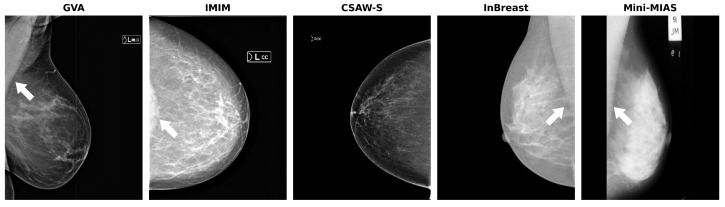
Sample images for each dataset. The white arrows indicate the pectoral muscle. Note the presence of the pectoral muscle in the cranio-caudal (CC) view of the IMIM dataset (second image).

A summary of the datasets is presented in Table 1, showing the number of images per view available in each split. Examples for each dataset are shown in Figure 1.

**Figure 2:**
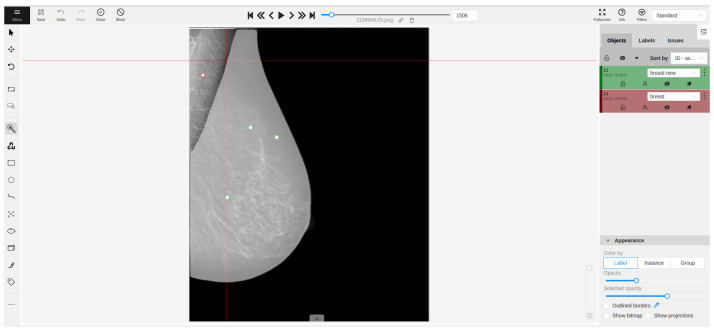
Main screen of CVAT, v2.5.1. Interaction with SAM is demonstrated through labeling with three positive (green) points and one negative (red) point.

In the proprietary datasets, ground truth labels were generated by two expert radiologists using the Segment Anything Model (SAM) within CVAT (Computer Vision Annotation Tool), v2.5.1. Consequently, the experts were able to use prompting points to select only the breast area, thereby excluding all unwanted regions. When the SAM failed to provide accurate delineation, conventional CVAT tools were employed for refinement. An example of a labeled mammogram in CVAT is depicted in Figure 2. For the public datasets, the labels were directly extracted from the previously mentioned references.

### 2.2. Models

In this section, we describe the different segmentation methods that we used to compare with our proposed SAM-breast model.

#### 2.2.1. Thresholding

Thresholding is a simple yet effective method for image segmentation. This technique assigns pixels to the foreground or background based on whether their intensity is above or below a certain threshold. Thresholding methods are widely employed in the delineation of breast boundaries [8].

The thresholding method that we implemented was presented in our previous work and includes two steps: breast detection and pectoral muscle exclusion [9].

In the breast detection step, an iterative algorithm based on connected components is used to obtain the gray level threshold that distinguishes the breast from the background. This is performed by considering the most frequent pixel value as the background and determining a range of possible breast thresholds from all unique values in the image. The image is then binarized using the first possible threshold before applying the scan-plus-array-based union-find (SAUF) algorithm. This process continues until only two homogeneous components are detected.

The pectoral muscle exclusion step assumes that the pectoral muscle appears as a triangle in one of the top corners of the image. An algorithm based on negative gradient changes is implemented, which requires the breast image to be left-oriented. A Gaussian filter with σ=3 and a 50-pixel moving window is applied to smooth edges and remove isolated bright pixels. The muscle border, being well-defined, tends to be the last remaining part after the smoothing process. A polygon is iteratively built to enclose the exclusion area by selecting the pixel with the lowest gradient every 50 rows until the column of the selected pixel is close enough to the left image border. The first pixel from the top left corner is taken as the vertex that closes the polygon.

#### 2.2.2. MedSegDiff

MedSegDiff-V2 is a novel transformer-based diffusion framework proposed for medical image segmentation. It integrates two cutting-edge techniques, the Diffusion Probabilistic Model (DPM) and vision transformer mechanisms. These techniques, which have been previously applied independently in the field, are combined in MedSegDiff-V2 to harness their collective strengths [24].

We trained MedSegDiff-V2 with the code provided by the original authors using the suggested hyperparameters (https://github.com/KidsWithTokens/MedSegDiff (accessed on 10 January 2024)). We processed the test datasets using five distinct embeddings. The final segmentation mask was then obtained by applying a threshold of 0.5.

#### 2.2.3. SAM

The Segment Anything Model (SAM) is a foundational model for image segmentation. It is designed to handle the promptable segmentation task while addressing real-world constraints. The SAM’s architecture consists of three primary modules: an image encoder, a prompt encoder, and a mask decoder. A single image embedding is produced by the image encoder, while different prompt encoding modules are specifically designed for efficient encoding of various prompt modes. Combining the image embedding with prompt encodings, a lightweight decoder then generates the segmentation masks [10].

In our evaluations, we utilized the ViT-H version of the original SAM. This model was employed with three prompting points, which were randomly placed across the ground segmentation masks in the test set.

#### 2.2.4. SAM-Adapter

Adapters, first used in Natural Language Processing (NLP), allow for efficient fine-tuning of large pre-trained models for specific tasks. Adaptation requires less than 5% of total parameters to be learned, enables quick updates, and is effective not only in NLP but also in computer vision. The Medical SAM Adapter, proposed by Wu et al. [17], is a specialized model designed to adapt the SAM architecture for medical image segmentation. It retains the pre-trained SAM parameters and introduces adapter modules at specific points in the architecture. The adapter module is a bottleneck model that uses a down-projection to compress the embedding to a smaller dimension and an up-projection to expand it back to its original size. The SAM encoder uses two adapters for each ViT block, positioned to optimize the multi-head attention and MLP layer. The SAM decoder utilizes three adapters for each ViT block, with modifications to incorporate prompt information, adjust the MLP-enhanced embedding, and handle the residue connection of the image embedding-to-prompt cross-attention. The final output is produced after adaptation, residue connection, and layer normalization.

We trained the Medical SAM Adapter with the code provided by the original authors (https://github.com/KidsWithTokens/Medical-SAM-Adapter (accessed on 23 November 2023)), using the training strategy described below. At inference, we used three prompting points randomly placed across the ground segmentation masks.

#### 2.2.5. SAM-Breast

We propose the SAM-breast model, which is also based on the original SAM architecture but with modifications to enhance breast delineation. In our model, we chose to freeze the weights of the image encoder. This decision was influenced by the considerable scale of the SAM, particularly since the encoder represents a major portion of the model’s weights. Modifying both the encoder and decoder not only would demand extensive hardware resources but has also been shown to compromise segmentation performance.

**Figure 3:**
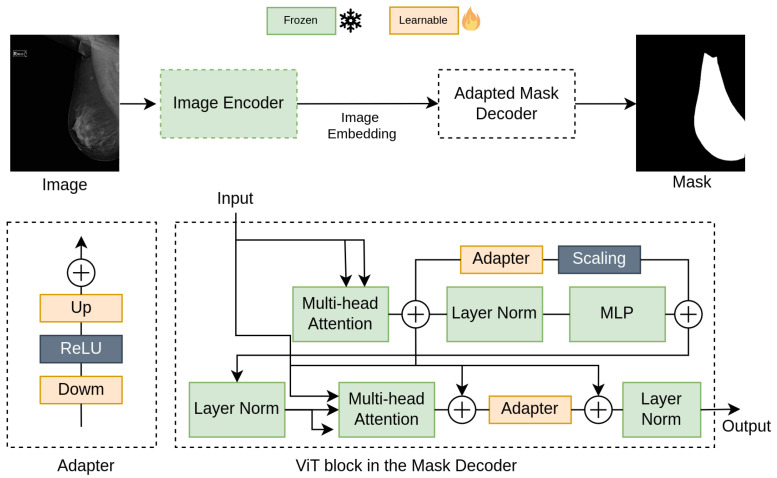
The architecture of SAM-breast. The encoder and layers of the mask decoder are frozen. Only the adapters incorporated in each ViT block are learned during training.

We eliminated the prompt encoder, thus removing the need for prompts during both the training and inference phases. The SAM decoder uses both prompt tokens and image embeddings, along with trainable output tokens for generating masks and predicting mask confidence. These output tokens, including foreground and background mask tokens, are combined with prompt tokens to form auxiliary embeddings. The decoder employs a two-way attention module that performs self-attention and cross-attention between tokens and the image embedding. The image embedding is then upscaled and combined with the foreground mask token to produce the mask. Following the method proposed by Hu et al., 2023 [15], we removed the prompt token from the auxiliary embeddings, making it non-promptable. As our objective is solely to predict the breast mask, we did not duplicate the embeddings as conducted in the original paper. Additionally, rather than training the entire mask decoder, we integrated two adapters for each ViT block, following the description by Wu et al., 2023 [17]. Consequently, we fine-tuned only the adapters within the mask decoder while still leveraging the original pre-trained weights. The architecture of SAM-breast is illustrated in Figure 3.

#### 2.2.6. Training Strategy

The following training strategy was implemented for the SAM-adapter and SAM-breast models.

The algorithms were implemented in Pytorch and trained for a maximum of 50 epochs. The epoch with the lowest validation loss was saved and used for test predictions. All the models were trained on an NVIDIA Tesla V100 (Santa Clara, CA, USA) using a batch size of 4 and the Adam optimizer with default parameters. We chose an initial learning rate of $1\times 10^{-5}$, which was divided by 2 whenever the validation loss did not decrease by more than 1% in one epoch. Two types of loss functions are normally used to train segmentation models: Binary Cross-Entropy (BCE) and the Dice Coefficient [25]. In our preliminary testings, we determined that the Dice loss had better convergence and achieved better results for this specific task.

The input images were resized to 1024×1024 pixels. To enhance the model’s generalization capabilities across a wide spectrum of mammograms, we randomly performed on-the-fly data augmentation during training. This process was specifically designed to introduce variations in image intensities and contrast, thereby exposing the model to a broader range of image variations during training. The augmentation process incorporated several transformations. These included random flipping and cropping of images, histogram shifting, and contrast adjustment within a specified range. Additionally, Gaussian noise was added to the images, and the intensity of the images was randomly scaled and shifted. These transformations were carefully chosen to simulate all possible cases that the model may encounter, thereby enhancing its robustness and predictive accuracy.

### 2.3. Evaluation

To measure the performance of the models, we selected a set of segmentation metrics commonly used for the evaluation of medical image segmentation [26] and used the implementations of the Monai library [27]. The selected metrics are as follows:

- Dice Similarity Coefficient (DSC): The DSC is a widely used metric for assessing the similarity between the predicted segmentation and the ground truth. It calculates the ratio of twice the intersection of the two segmentations to the sum of the number of pixels in each segmentation. We express this metric as a percentage, ranging from 0 to 100%, with a higher value signifying a superior model performance. The DSC can be expressed as:
  (1) $$\begin{array}{c} \begin{array}{c} DSC\left(X,Y\right)=\frac{2|X\cap Y|}{\left|X|+|Y\right|} \end{array} \end{array}$$
  where *X* and *Y* denote the binary segmentation and the ground truth, respectively.
- Intersection over Union (IoU): The IoU, also known as the Jaccard index, measures the overlap between two segmentations. While it bears resemblance to the DSC, its calculation method differs. The IoU can provide a more stringent measure of segmentation performance than the DSC because it penalizes false positives and false negatives equally. It also ranges from 0 to 100%, with higher values indicating better performance. The formula for IoU is given by:
  (2) $$\begin{array}{c} \begin{array}{c} IoU\left(X,Y\right)=\frac{\left|X\cap Y\right|}{\left|X\cup Y\right|} \end{array} \end{array}$$
- Hausdorff Distance (HD): The HD is a metric based on spatial distance. It calculates the maximum distance from a point in one set to the closest point in the other set, allowing for the scoring of localization similarity by focusing on contour delineation. Compared to the overlapping metrics (DSC and IoU), the HD is more sensitive to the boundary. The HD is measured in pixels, with lower values indicating better performance. The formula is given by:
  (3) $$\begin{array}{c} \begin{array}{c} HD\left(X,Y\right)=\max\left\{\max_{x\in X}\min_{y\in Y}d\left(x,y\right),\max_{y\in Y}\min_{x\in X}d\left(x,y\right)\right\} \end{array} \end{array}$$
  where d(x,y) represents the distance between points *x* and *y*.
- Average Surface Distance (ASD): This is another metric used to evaluate the quality of image segmentation. It calculates the average distance from each point on the boundary of the predicted segmentation to the closest point on the boundary of the ground truth segmentation. The ASD is less sensitive to outliers than HD, as it considers the average performance over all points, not just the worst one. It is also measured in pixels, with lower values indicating better performance. The formula for the ASD is given by:
  (4) $$\begin{array}{c} \begin{array}{c} ASD\left(X,Y\right)=\frac{1}{\left|X\right|}\sum_{x\in X}\min_{y\in Y}d\left(x,y\right) \end{array} \end{array}$$
  where |X| is the measure of the surface *X* and d(x,y) is the Euclidean distance between points *x* and *y*.

## 3. Results

**Figure 4:**
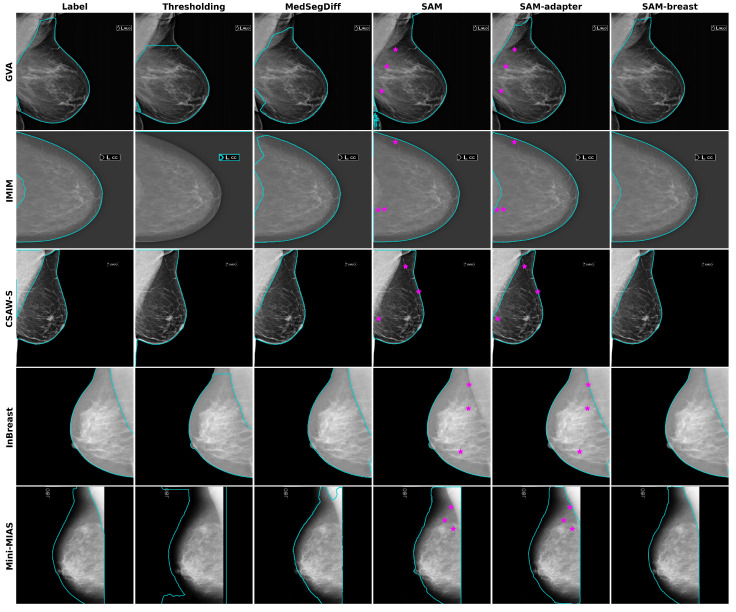
Representative examples for each dataset and model tested. The prompting points used for the SAM and SAM-adapter models are also indicated as pink stars. Note the correct exclusion of the pectoral muscle in the CC view image (second row).

All the models described were tested on the different datasets, and a summary of the results is presented in Table 2. Based on these results, our proposed SAM-breast model performs the best for most metrics across all datasets. The only exception is the MedSegDiff model, which shows the best distance metrics (HD and ASD). However, when considering the overall results and visual assessments, SAM-breast shows superior performance. Segmentation examples from each model and dataset are depicted in Figure 4.

**Figure 5:**
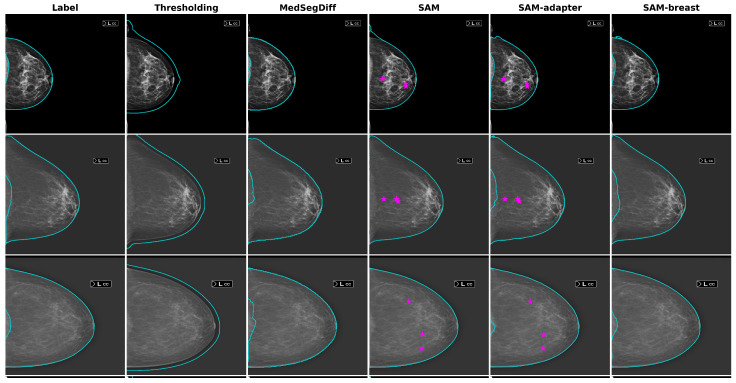
Cranio-caudal (CC) examples where the pectoral muscle is present. The prompting points used for the SAM and SAM-adapter models are also indicated as pink stars.

In Table 3, we present the results for the images in CC views. This table demonstrates that all models perform exceptionally well in CC views. However, the metrics do not account for the small percentage of pectoral muscle present in the CC views. This is further illustrated in Figure 5, where it is evident that the proposed SAM-breast model successfully excludes the pectoral muscle, even in these images.

The MLO views are significantly more complex due to the presence of the pectoral muscle. Consequently, Table 4 displays lower metrics primarily for the thresholding method and the original SAM.

As outlined earlier, the proprietary datasets utilized in our study were sourced from various centers and acquisition devices. Table 5 presents the results corresponding to each distinct device within the proprietary datasets. This analysis confirms that the metrics obtained are consistent across different acquisition devices.

## 4. Discussion

We have presented the SAM-breast model for delineating the breast area in digital mammograms. Our results demonstrate the superior performance of the proposed model compared to traditional methods and other deep learning models.

The traditional thresholding technique used in this study has proven to be successful in a large number of scenarios. However, it does not perform as expected when the image contains the abdomen or when the pectoral muscle is included in the CC views [9]. The SAM-breast model effectively addresses this issue by excluding the pectoral muscle in both the MLO and CC views.

The effectiveness of the MedSegDiff model has been thoroughly evaluated across a broad range of medical image segmentation tasks. Specifically, it has been tested on 20 tasks involving different image modalities. This extensive testing underscores the model’s versatility and adaptability to various types of medical imaging data. It has demonstrated a significant improvement in segmentation accuracy compared to previous image segmentation models [24]. However, in our tests, MedSegDiff did not perform as well as SAM-breast. One drawback of the MedSegDiff model is that it takes about two minutes on a single GPU to produce one prediction, while SAM-breast only takes about one second.

The original SAM can generate segmentation masks with impressive speed and quality in most natural images [10]. However, its performance was not optimal on the different mammogram test sets. These results reinforce the necessity of adapting the SAM for specific tasks, especially for medical images.

To compare our results with previously published methodologies, we calculated the accuracy of the SAM-breast model for the Mini-MIAS and InBreast datasets. This was performed in light of the fact that accuracy is frequently employed as an evaluation metric in the majority of studies conducted on these datasets. We present a comparison with this metric in Table 6. The most recent study that aligns with our work is by Zhou et al. [28]. They implemented the Deeplab v3+ model, incorporating preprocessing steps such as noise suppression and contrast enhancement, which resulted in a DSC of 98.48% and IoU of 97.39% for the Mini-MIAS dataset. These values are slightly higher than those achieved by our model on the same dataset (DSC 98.07% and IoU 96.29%). However, it is important to note that their model was trained and tested on different subsets of the Mini-MIAS dataset. In contrast, our proposed model was trained on a proprietary dataset, implying that the public datasets were not seen during the training phase. This is indicative of the robust generalization performance of our model. For example, Zhou et al. also evaluated their model independently on the entire InBreast dataset (comprising 410 images), achieving a DSC of 98.48% and an IoU of 97.14%, whereas our model, tested on 200 MLO images of the InBreast dataset, achieved a DSC of 99.27% and an IoU of 98.55%. Therefore, our model consistently delivers high performance, even when analyzing images with different resolutions and originating from various vendors. We would like to highlight that our approach did not involve any image preprocessing. Instead, our focus was on extensive data augmentation during the training phase. This was achieved by simulating various contrast and intensity variations, thereby enabling us to develop a model that exhibits robust performance across a wide range of scenarios.

## 5. Conclusions

In this study, we presented the SAM-breast model, a specialized adaptation of the SAM, designed for the precise segmentation of the breast region in mammographic imaging. Our findings indicate that the SAM-breast model proficiently delineates the breast area, effectively excluding the pectoral muscle across both MLO and, notably, CC views where its presence is less frequent. The robustness of our model was validated across diverse datasets sourced from various acquisition devices, showcasing consistent performance throughout. The success of the SAM-breast model underscores the versatility and adaptability of the SAM framework when customized for specific medical imaging tasks, such as the segmentation of breast tissue in mammograms. This advancement holds significant promise for enhancing the accuracy and efficiency of breast cancer screening protocols.

## Figures and Tables

**Table 1:** Summary of the datasets used for training and testing.

Dataset	Split	#MLO	#CC	Total
GVA	Train	1052	1190	2242
GVA	Validation	111	139	250
GVA	Test	395	449	844
IMIM	Test	0	881	881
CSAW-S	Test	126	144	270
InBreast	Test	200	0	200
Mini-MIAS	Test	322	0	322

MLO: medio lateral-oblique. CC: cranio-caudal.

**Table 2:** A summary of the results for each dataset.

Dataset	Model	DSC	IoU	HD	ASD
	Thresholding	95.81 ± 4.80	92.31 ± 7.58	101.87 ± 91.44	10.21 ± 16.66
	MedSegDiff	98.40 ± 1.18	96.88 ± 2.17	**5.96 ± 7.58**	**0.96 ± 0.89**
GVA	SAM	95.32 ± 5.73	91.60 ± 9.80	111.70 ± 130.12	19.78 ± 40.17
	SAM-adapter	99.21 ± 0.87	98.45 ± 1.65	20.88 ± 27.95	1.85 ± 2.21
	SAM-breast	**99.37 ± 0.86**	**98.76 ± 1.61**	18.72 ± 30.19	1.64 ± 2.95
	Thresholding	97.14 ± 8.00	95.32 ± 11.24	58.20 ± 100.52	13.88 ± 45.56
	MedSegDiff	98.91 ± 1.45	97.87 ± 2.59	**5.77 ± 9.14**	**0.77 ± 1.12**
IMIM	SAM	99.22 ± 1.04	98.47 ± 1.95	42.10 ± 69.11	4.14 ± 15.83
	SAM-adapter	99.49 ± 0.59	98.98 ± 1.14	18.20 ± 21.84	1.45 ± 1.90
	SAM-breast	**99.54 ± 0.52**	**99.10 ± 1**	15.09 ± 19.31	1.27 ± 1.74
	Thresholding	92.48 ± 8.36	86.94 ± 12.30	152.50 ± 104.63	23.04 ± 21.50
	MedSegDiff	97.19 ± 3.98	94.80 ± 6.74	**15.26 ± 23.05**	2.83 ± 7.06
CSAW-S	SAM	94.26 ± 6.70	89.85 ± 11.10	129.10 ± 126.87	20.39 ± 29.58
	SAM-adapter	98.72 ± 1.41	97.52 ± 2.60	58.15 ± 75.96	3.21 ± 3.81
	SAM-breast	**99.10 ± 0.96**	**98.22 ± 1.83**	51.89 ± 75.16	**2.54 ± 3.22**
	Thresholding	94.70 ± 2.74	90.06 ± 4.77	146.33 ± 72.53	12.66 ± 7.02
	MedSegDiff	98.37 ± 1.23	96.82 ± 2.30	**7.56 ± 8.27**	**1.10 ± 0.88**
InBreast	SAM	92.04 ± 5.16	85.65 ± 8.48	170.89 ± 71.21	28.77 ± 16.92
	SAM-adapter	99.05 ± 1.18	98.13 ± 2.15	30.98 ± 36.24	2.59 ± 3.29
	SAM-breast	**99.27 ± 0.85**	**98.55 ± 1.62**	25.37 ± 32.76	2.08 ± 2.42
	Thresholding	81.30 ± 7.36	69.11 ± 10.05	187.58 ± 57.46	52.02 ± 13.64
	MedSegDiff	87.81 ± 6.59	78.85 ± 9.98	46.18 ± 16.24	10.79 ± 5.38
Mini-MIAS	SAM	89.04 ± 6.24	80.78 ± 9.61	199.71 ± 79.20	40.45 ± 26.70
	SAM-adapter	95.35 ± 4.37	91.43 ± 7.43	81.66 ± 60.26	13.68 ± 12.47
	SAM-breast	**98.07 ± 2.11**	**96.29 ± 3.82**	**46.06 ± 41.70**	**6.55 ± 8.13**

DSC: Dice Similarity Coefficient. IoU: Intersection over Union. HD: Hausdorff Distance. ASD: Average Surface Distance. Values are shown as mean ± standard deviation. The best result for each evaluation metric and dataset is highlighted in bold.

**Table 3:** Results for images in CC view.

Dataset	Model	DSC	IoU	HD	ASD
	Thresholding	98.17 ± 3.65	96.59 ± 4.88	42.57 ± 56.90	6.14 ± 19.33
	MedSegDiff	98.73 ± 0.68	97.49 ± 1.31	**4.70 ± 6.96**	**0.77 ± 0.50**
GVA	SAM	99.25 ± 1.39	98.54 ± 2.42	45.80 ± 104.89	6.73 ± 36.58
	SAM-adapter	99.49 ± 0.48	98.99 ± 0.94	15.02 ± 24.52	1.21 ± 1.31
	SAM-breast	**99.60 ± 0.42**	**99.21 ± 0.82**	12.96 ± 23.97	0.96 ± 1.08
	Thresholding	97.14 ± 8.00	95.32 ± 11.24	58.20 ± 100.52	13.88 ± 45.56
	MedSegDiff	98.91 ± 1.45	97.87 ± 2.59	**5.77 ± 9.14**	**0.77 ± 1.12**
IMIM	SAM	99.22 ± 1.04	98.47 ± 1.95	42.10 ± 69.11	4.14 ± 15.83
	SAM-adapter	99.49 ± 0.59	98.98 ± 1.14	18.20 ± 21.84	1.45 ± 1.90
	SAM-breast	**99.54 ± 0.52**	**99.10 ± 1**	15.09 ± 19.31	1.27 ± 1.74
	Thresholding	89.72 ± 9.14	82.37 ± 12.57	193.76 ± 99.24	33.17 ± 21.63
	MedSegDiff	97.18 ± 4.00	94.76 ± 6.66	**16.13 ± 21.39**	**2.59 ± 5.91**
CSAW-S	SAM	91.59 ± 7.05	85.21 ± 11.37	167.77 ± 108.24	26.39 ± 22.34
	SAM-adapter	98.56 ± 1.39	97.20 ± 2.54	79.14 ± 86.93	3.53 ± 3.71
	SAM-breast	**98.99 ± 0.80**	**98.02 ± 1.53**	71.05 ± 84.74	2.86 ± 3.23

DSC: Dice Similarity Coefficient. IoU: Intersection over Union. HD: Hausdorff Distance. ASD: Average Surface Distance. Values are shown as mean ± standard deviation. The best result for each evaluation metric and dataset is highlighted in bold.

**Table 4:** Results for images in MLO view.

Dataset	Model	DSC	IoU	HD	ASD
	Thresholding	93.13 ± 4.54	87.44 ± 7.15	169.28 ± 75.13	14.84 ± 11.33
	MedSegDiff	98.03 ± 1.49	96.18 ± 2.69	**7.38 ± 8.01**	**1.17 ± 1.15**
GVA	SAM	90.86 ± 5.53	83.70 ± 9.03	186.62 ± 114.64	34.62 ± 38.94
	SAM-adapter	98.90 ± 1.08	97.84 ± 2.03	27.56 ± 30.07	2.57 ± 2.74
	SAM-breast	**99.11 ± 1.11**	**98.25 ± 2.07**	25.89 ± 34.87	2.40 ± 4.02
	Thresholding	95.62 ± 6.01	92.16 ± 9.66	105.36 ± 89.95	11.47 ± 14.36
	MedSegDiff	97.22 ± 3.97	94.84 ± 6.86	**14.25 ± 24.87**	3.11 ± 8.19
CSAW-S	SAM	97.32 ± 4.68	95.15 ± 8.03	84.91 ± 132.46	13.53 ± 34.98
	SAM-adapter	98.91 ± 1.41	97.88 ± 2.62	34.15 ± 51.81	2.83 ± 3.90
	SAM-breast	**99.21 ± 1.11**	**98.46 ± 2.09**	30 ± 55.11	**2.17 ± 3.17**
	Thresholding	94.70 ± 2.74	90.06 ± 4.77	146.33 ± 72.53	12.66 ± 7.02
	MedSegDiff	98.37 ± 1.23	96.82 ± 2.30	**7.56 ± 8.27**	**1.10 ± 0.88**
InBreast	SAM	92.04 ± 5.16	85.65 ± 8.48	170.89 ± 71.21	28.77 ± 16.92
	SAM-adapter	99.05 ± 1.18	98.13 ± 2.15	30.98 ± 36.24	2.59 ± 3.29
	SAM-breast	**99.27 ± 0.85**	**98.55 ± 1.62**	25.37 ± 32.76	2.08 ± 2.42
	Thresholding	81.30 ± 7.36	69.11 ± 10.05	187.58 ± 57.46	52.02 ± 13.64
	MedSegDiff	87.81 ± 6.59	78.85 ± 9.98	46.18 ± 16.24	10.79 ± 5.38
Mini-MIAS	SAM	89.04 ± 6.24	80.78 ± 9.61	199.71 ± 79.20	40.45 ± 26.70
	SAM-adapter	95.34 ± 4.38	91.41 ± 7.44	81.86 ± 60.24	13.71 ± 12.47
	SAM-breast	**98.07 ± 2.11**	**96.29 ± 3.82**	**46.06 ± 41.10**	**6.55 ± 8.13**

DSC: Dice Similarity Coefficient. IoU: Intersection over Union. HD: Hausdorff Distance. ASD: Average Surface Distance. Values are shown as mean ± standard deviation. The best result for each evaluation metric and dataset is highlighted in bold.

**Table 5:** Results for different devices using the proposed SAM-breast model.

Dataset	Device	DSC	IoU	HD	ASD
	01-FUJIFILM	99.12 ± 1.64	98.30 ± 2.98	19.98 ± 26.20	1.94 ± 3.07
	02-FUJIFILM	99.59 ± 0.44	99.19 ± 0.86	10.41 ± 14.68	0.96 ± 1.07
	04-IMS Giotto	99.37 ± 1.08	98.78 ± 2.03	12.85 ± 10.22	1.25 ± 1.32
	05-FUJIFILM	99.55 ± 0.34	99.10 ± 0.67	13.98 ± 26.48	1.05 ± 1.06
	07-HOLOGIC	99.27 ± 1.12	98.58 ± 2.14	27.21 ± 62.35	3.95 ± 10.97
GVA	10-SIEMENS	99.06 ± 1.26	98.16 ± 2.33	27.94 ± 33.87	2.48 ± 4.33
	11-FUJIFILM	99.44 ± 0.54	98.90 ± 1.05	14.40 ± 24.22	1.37 ± 1.94
	13-FUJIFILM	99.43 ± 0.84	98.87 ± 1.59	19.72 ± 40.30	1.54 ± 3.47
	18-IMS Giotto	99.14 ± 0.67	98.30 ± 1.30	19.43 ± 22.83	2.16 ± 2.02
	20-Unknown	99.13 ± 0.73	98.29 ± 1.41	28.27 ± 41.29	2.04 ± 2.34
	99-GE	99.46 ± 0.38	98.93 ± 0.76	17.72 ± 32.02	1.52 ± 1.10
	FUJIFILM	99.34 ± 0.47	98.69 ± 0.92	16.25 ± 18.37	1.86 ± 1.55
IMIM	GE	99.28 ± 0.71	98.59 ± 1.38	23.98 ± 30.83	1.97 ± 2.91
	HOLOGIC	99.31 ± 0.55	98.64 ± 1.05	14.70 ± 17.75	1.71 ± 1.61

DSC: Dice Similarity Coefficient. IoU: Intersection over Union. HD: Hausdorff Distance. ASD: Average Surface Distance. Values are shown as mean ± standard deviation. FUJIFILM (Tokyo, Japan), IMS Giotto (Sasso Marconi, Italy), HOLOGIC (Marlborough, MA, USA), SIEMENS (Munich, Germany), GE (Boston, MA, USA).

**Table 6:** A comparison of the accuracy of various published methods.

Dataset	Method	Year	Images	Accuracy
	Lbachir et al. [29]	2017	40	90%
	Taghanaki et al. [30]	2017	197	96%
InBreast	Rahman et al. [31]	2019	200	94.50%
	Zhou et al. [28]	2022	410	99.12%
	SAM-breast (proposed)	2024	200	99.56%
	Lbchir et al. [29]	2017	322	98.75%
	Taghanaki et al. [30]	2017	322	95%
Mini-MIAS	Rahman et al. [31]	2019	200	97.50%
	Zhou et al. [28]	2022	102	98.98%
	SAM-breast (proposed)	2024	322	98.76%

## Data Availability

The public datasets used in this study can be found at CSAW-S https://zenodo.org/records/4030660#.X2HD15MzZhE (accessed on 7 August 2023), InBreast https://www.kaggle.com/datasets/tommyngx/inbreast2012 (accessed on 30 October 2023), Mini-MIAS http://peipa.essex.ac.uk/info/mias.html (accessed on 11 January 2024), https://zenodo.org/records/10149914 (accessed on 11 January 2024).
